# Morphological Structure, Rheological Behavior, Mechanical Properties and Sound Insulation Performance of Thermoplastic Rubber Composites Reinforced by Different Inorganic Fillers

**DOI:** 10.3390/polym10030276

**Published:** 2018-03-07

**Authors:** Yanpei Fei, Wei Fang, Mingqiang Zhong, Jiangming Jin, Pin Fan, Jingtao Yang, Zhengdong Fei, Feng Chen, Tairong Kuang

**Affiliations:** 1 College of Materials Science and Engineering, Zhejiang University of Technology, Hangzhou 310014, China; 201101391305@zjut.edu.cn (Y.F.); 2111625018@zjut.edu.cn (W.F.); zhongmq@zjut.edu.cn (M.Z.); fanping@zjut.edu.cn (P.F.); yangjt@zjut.edu.cn (J.Y.); feizd@zjut.edu.cn (Z.F.); 2 College of Mechanical Engineering, Zhejiang University of Technology, Hangzhou 310014, China; 3 Key Laboratory of Polymer Processing Engineering of Ministry of Education, South China University of Technology, Guangzhou 510630, China; 4 State Key Laboratory of Molecular Engineering of Polymers, Fudan University, Shanghai 200433, China

**Keywords:** thermoplastic rubber, composites, sound insulation property, mechanical property, viscous behavior

## Abstract

The application area of a sound insulation material is highly dependent on the technology adopted for its processing. In this study, thermoplastic rubber (TPR, polypropylene/ethylene propylene diene monomer) composites were simply prepared via an extrusion method. Two microscale particles, CaCO_3_ and hollow glass microspheres (HGW) were chosen to not only enhance the sound insulation but also reinforced the mechanical properties. Meanwhile, the processing capability of composites was confirmed. SEM images showed that the CaCO_3_ was uniformly dispersed in TPR matrix with ~3 μm scale aggregates, while the HGM was slightly aggregated to ~13 μm scale. The heterogeneous dispersion of micro-scale fillers strongly affected the sound transmission loss (STL) value of composites. The STL values of TPR composites with 40 wt % CaCO_3_ and 20 wt % HGM composites were about 12 dB and 7 dB higher than that of pure TPR sample, respectively. The improved sound insulation performances of the composites have been attributed to the enhanced reflection and dissipate sound energy in the heterogeneous composite. Moreover, the mechanical properties were also enhanced. The discontinued sound impedance and reinforced stiffness were considered as crucial for the sound insulation.

## 1. Introduction

Nowadays, noise pollution becomes much more serious with the rapid development of industry and transportation. Therefore the technique that the preparation of damping and noise reduction materials has attracted many attentions for environment and health safety [1]. Compared with the traditional metal materials, polymer materials as a great advantage in noise control engineering due to its superior performance, such as excellent viscoelasticity and good process-capability [2,3,4]. However, a number of polymer materials cannot meet the requirements on strength, toughness and other mechanical properties.

Polymeric materials with micro/nano-structures have attracted increasing interest from both academic and industrial field. The micro- or nano-structure in polymeric materials can give excellent physical properties and multifunctional applications [5,6,7,8,9]. Therefore, hierarchical scale structure design of polymeric materials is regarded as one of the important route to achieve outstanding performance [10,11,12]. To achieve outstanding sound-insulation property, many efforts were contributed for fabrication of hierarchical scale structure. A number of soundproof composites has been developed likes wood-waste tire rubber composite [13], inorganic particles/polymer composites and nano-composites, including polypropylene/CaCO_3_ [14], resin/hollow glass bead [15], poly(vinyl chloride)/mica [16], rubber/carbon nanotube [17], polyvinylpyrrolidone/graphene oxide [18], poly(vinyl acetate) mesoporous carbon [19], and so on. Besides, the porous structure [20] and honeycomb structure [21,22] can also enhance the sound insulation performance. But it is hard to achieve both high soundproof and mechanical properties of traditional polymer composites. Very recently, Guo’s group developed a bilayer and multilayer plate structure, which could efficaciously attenuate acoustic energy by employing viscoelastic polymers as an interlayer of sandwich structure to increase sound transmission loss due to their high damping properties [23]. Liang reported that polymer foams in a sandwich structure could also enhance the soundproof property owing to the viscoelastic air cells and increased sound wave propagation routine [24]. However, special die is designed to achieve the multilayered distribution of fillers in polymer matrices and the multilayer co-extrusion technique is necessary; as a result, the process has complicated and extra cost has charged. Hereby, it is prominent to produce excellent soundproof polymer composites with enhanced mechanical properties by means of common feasible processing technologies.

As a very important damping material, thermoplastic rubber (TPR) has broadly applied in transportation, architecture and electronic products [25]. For large-scale and low-cost processing, injection molding and extrusion are most stable processing methods, however, the soundproof, and mechanical properties cannot simultaneously meet strict requirements. Previously, our group has confirmed that adding either CaCO_3_ or hollow glass microspheres (HGM) could greatly increase the stiffness of polymer, which is beneficial to enhance the sound wave refraction, scatting and reflection (Figure S1 and Table S1). In addition, due to the great difference of elastic moduli between the polymer and inorganic fillers, the sound wave can easily dissipate on the interface. For the sake of excellent damping property of TPR, we choose the polypropylene/ethylene propylene diene monomer (PP/EPDM) composite, compounding with micro-scale CaCO_3_ and HGM particles. The sound insulation of the composites were markedly enhanced especially in a range of 50–1500 Hz, meanwhile the mechanical properties were simultaneously improved.

## 2. Experiment

### 2.1. Materials and Sample Preparation

Ethylene propylene diene monomer (EPDM) was grade 725P and obtained from the Dow Chemical Company. Polypropylene (PP) was grade T30s and purchased from the Daqing Petrochem. Co., Ltd. (Daqing, China). The tacticity of PP is 96.5%. Prior to the melt extrusion, CaCO_3_ and HGM particles were dried in vacuum for 1 h, then mixed in a mixing machine 1 h respectively, 2% content tetrabutyl titanate was added and mixed together for 1 h, and finally dried in vacuum overnight. The TPR samples were prepared in the HAAKE™ Rheomix OS Lab Mixer at 180 °C and 60 r/min for 10 min. The detailed specifications of all samples are summarized in Table 1.

### 2.2. Morphology Characterization

The morphology of pure TPR, the inorganic fillers and the dispersion of inorganic particles in the composites were characterized by using a scanning electron microscopy (SEM type S-4700, JEOL, Akishima-shi, Japan). The fractured surface of TPR composites was obtained by immersing samples in liquid nitrogen and spayed with gold before SEM examination. In order to verify the exact dispersion of inorganic fillers, the fracture surface of TPR composites were etched by hydrochloride acid (10 wt %) to remove CaCO_3_ particles, or hydrofluoric acid (1 wt %) to remove HGM particles.

### 2.3. Rheology and Mechanical Properties

The rheological properties of the blends were studied using a capillary rheometer (Rosand RH7, Malvern, Worcestershire, UK) into which the material was loaded by a plunger through a capillary. The load in the plunger provided the total pressure drop in the barrel and capillary. The rheological experiments were carried out at 180 °C, using a L/R = 19.33 capillary. The corrections suggested by Bagley [26] were used considering the data from the two capillary dies.

Dynamic mechanical thermal analysis was conducted using dynamic thermal mechanical analyzer (DMA type Q-800 TA Instruments, New Castle, DE, USA). The sample size is cut to small plate with a scale of 30 × 10 × 2 mm. The mode is single cantilever. The temperature range is ranging from −60 to 20 °C. The heating rate and frequency are set to 3 °C/min and 1 Hz, respectively.

Tensile specimens were obtained from hot-press to dumbbell-shaped samples. The tensile testing used an Instron 5996 tension machine (Instron Corporation, Norwood, MA, USA) at 23 °C, according to ASTMD 638, at the displacement rate of 50 mm/min. At least five specimens for each sample were tested and the average value was calculated. Impact specimens were obtained from hot-press to notched impact samples. The impact testing used a Ceast 9050 impact testing machine (Instron Corporation, Norwood, MA, USA) at 23 °C, according to GB 1843-2008. At least five specimens for each sample were tested and the average value was calculated.

### 2.4. Sound Insulation Property

The sound transmission loss (STL), representing the soundproof efficiency, is defined to the logarithmic ratio of the incident acoustic power to transmitted acoustic power. STL value was tested using a Bruel and Kjaer, four-microphone small standing wave tube (Type: 4206-T) as shown in Figure S2. The effective sound wave was measured in the range from 500 to 6000 Hz at 25 °C. The thickness of all samples was 5 mm. Detailed theory is summarized in the supplementary material. The theoretical STL value was calculated by Equation (1) using a transfer function method

STL = −20 × log|*t*| (1)

where *t* is the ratio of the transmitting sound energy to the incident sound energy.

To investigate the acoustic impedance (*Z*), the stiffness (*S*) and surface density ($\bar{\rho }$) of the material can be calculated from the following equations, where *S*, *E*, *h*, *μ*, $\bar{\rho }$ and *ρ* are stiffness, modulus, thickness, Poisson ratio, surface density and density of the sample, respectively.

(2) $$S=\frac{1}{12}\times \frac{E\times h^{3}}{1-\mu^{2}}$$

(3) $$\bar{\rho }=\rho \times h$$

The acoustic impedance (*Z*) of the material is the product of sound speed (*C*) and the density (*ρ*) of the material, while the longitudinal wave speed (*C*) in solid can be calculated according to Equation (3), where *E*, *μ* and *ρ* are elastic modulus, Poisson ratio and density, respectively.

(4) $$C=\sqrt{\frac{E\times (1-\mu )}{\rho \times (1+\mu )\times (1-2\mu )}}$$

(5) Ζ=ρ×C

### 2.5. Density Test

The weight (*m*) of samples was measured by the electronic scales (FA1104N, Shanghai, China). The initial water volume (*V*_0_) and the volume (*V*) after the samples needled into the water were measured by the measuring cylinder. The density equals quality divided by the volume that was *V* minus *V*_0_. At least five specimens for each sample were tested and the average value was calculated.

(6) $$\rho =\frac{m}{V-V_{0}}$$

## 3. Results and Discussion

### 3.1. Viscous Behavior

**Figure 1:**
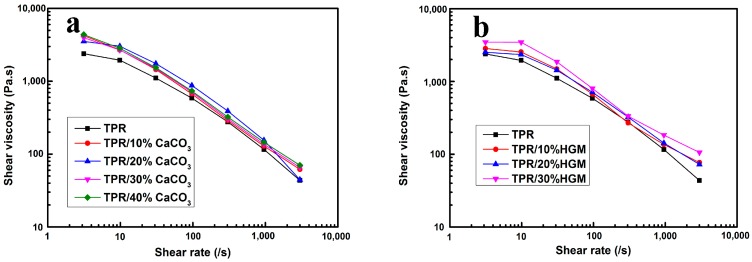
Shear viscosity versus shear rate of various (**a**) TPR/CaCO_3_ composites and (**b**) TPR/HGM composites. PR: Melt blending of PP with EPDM (PP/EPDM = 30/70); HGM: hollow glass microspheres.

Figure 1 shows effects of inorganic filler content on shear viscosity of TPR composites at 180 °C. The EPDM content of TPR matrix was chosen as 70 wt %, since there was almost one loss factor peak at −27 °C (Figure S3), representing the good compatibility of PP and EPDM. It is apparent that TPR and their composites showed typical shear-thinning behavior over the range of shear rates in that the shear viscosity decreased with an increase in shear rate. This behavior was attributed to the alignment or arrangement of the chain segments of polymers to the direction of the melt flow through capillary. Such behavior was reported for other polymeric systems containing TPR [27,28,29]. In general, the high value of viscosities at low shear rates would provide the integrity of the extrudate during extrusion, while the low viscosities at high shear rates caused low injection temperature and pressure as well as short time for injection cycle. Thus all composites in this study are suitable for processing by both extrusion and injection. The viscosity values of the blends increased with the increase of inorganic filler contents. These results were useful for optimizing the processing conditions of TPR composites would be quite different from that of neat TPR. For example, the processing capability of TPR composites whose viscosity was slight higher than neat TPR would be close to that of neat TPR.

### 3.2. Morphology

**Figure 2:**
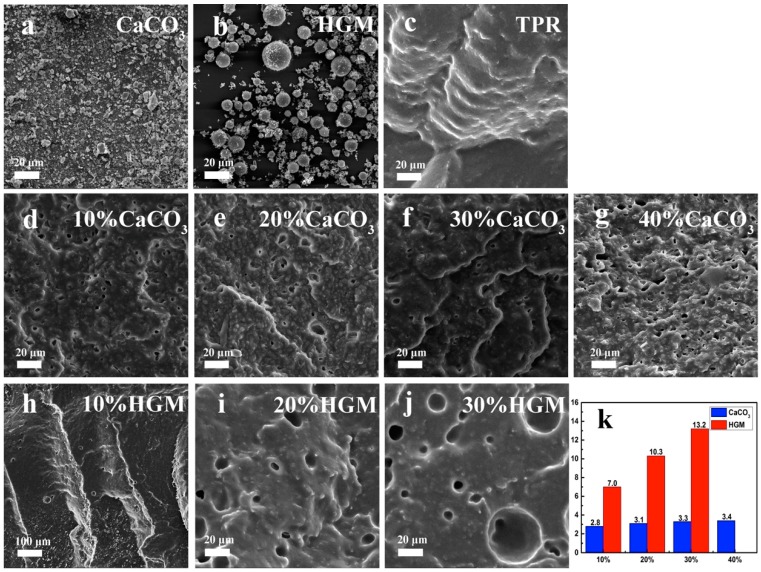
The morphology of inorganic particles and various TPR composites observed by SEM (the magnification ratio was 1000 times). (**a**) micro CaCO_3_; (**b**) HGM; (**c**) pure TPR; (**d**) TPR/10%CaCO_3_; (**e**) TPR/20%CaCO_3_; (**f**) TPR/30%CaCO_3_; (**g**) TPR/40%CaCO_3_; (**h**) TPR/10%HGM; (**i**) TPR/20%HGM; (**j**) TPR/30%HGM; (**k**) average pore size of different content of inorganic particles.

To study the dispersion of CaCO_3_ and HGM in the TPR matrix, the samples of various composites were fractured in liquid nitrogen. Thus, SEM could be applied to characterize the morphology of different composites. As shown in Figure 2a,b, the average size of modified CaCO_3_ and HGM micro-particle was 2.0 and 6.8 μm, respectively. Figure 2c shows the fracture surface of neat TPR matrix, indicating very good compatibility of PP and EPDM. Li [30] reports on the excellent dispersivity of Thermoplastic elastomer. As the content of CaCO_3_ micro-particles increased from 10 wt % to 40 wt %, though the composites exhibited slight aggregation of CaCO_3_ from 2.8 to 3.4 μm (Figure 2k); while the HGM was more likely to form orbicular agglomerations larger than 10 μm at the similar condition (Figure 2k). The good dispersion of inorganic fillers not only can stiffen the polymer matrix, but also can influence the movement of viscoelastic polymer domain.

### 3.3. Sound Insulation Property

**Figure 3:**
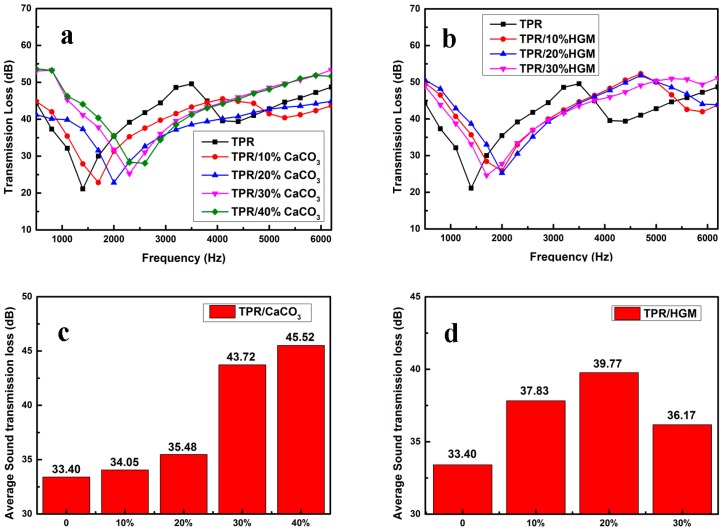
Sound insulation property of neat TPR and its composites: The STL (sound transmission loss) curves versus the sound frequency of (**a**) TPR/CaCO_3_ composites and (**b**) TPR/HGM composites; the average STL value of (**c**) TPR/CaCO_3_ composites and (**d**) TPR/HGM composites.

Generally, the sound wave was reflected by the interface and absorbed by the viscoelastic materials. In this work, frequencies ranging in 500 to 6000 Hz were selected to investigate the sound insulation property of TPR composites, due to the soundproofing efficiency of the material corresponds to the frequency of sound wave [13,31,32]. The dependence of sound transmission loss (STL) value was depicted in Figure 3. All samples exhibited similar trends of STL value influenced by increasing the sound wave frequency. Position of the first resonance frequency on the frequency scale can be used for following the increasing stiffness of the tested materials. It was shifting from lower frequencies in the case of virgin TPR to higher frequencies for micro-particle filled composites. This phenomenon quite differed with crystalline polymer based system, such as high-density polyethylene (HDPE)/CaCO_3_ hollow sphere composite [33], while similar results could be referred in multilayered polymer [23] or foam composites [34]. In comparison with neat TPR sample, the STL values of composite samples improved significantly with the addition of CaCO_3_ and HGM micro-particles in lower frequency zone (below the first resonance frequency). With increasing either CaCO_3_ or HGM content, the STL value of samples gradually enhanced. For comparison, the average STL values of all samples were plotted in Figure 3c,d. The average STL value of neat TPR was 33.40 dB, while the sound insulation property of composites was effectively improved. For example, the sample containing 30 wt % CaCO_3_ reached an enhanced STL value of 43.52 dB, which was 1.30 time of that of neat TPR. Compared to CaCO_3_-filled composite with the same filler content, HGM-filled samples showed better STL values below 30 wt % filler content. However, the STL value of the sample with higher HGM content decreased, possibly due to the propagation route of the sound wave would be chopped by aggregative fillers.

When the sound frequency is low, the composites can respond imitatively the vibration from the sound wave to make an equilibrium, and present obvious sensitivity of the transmission loss to the sound frequency (Figure 3). While in the case of high sound frequency, the composites cannot respond imitatively the vibration from the sound wave to make equilibrium, and present that the transmission loss increases slightly with an increase of the sound frequency.

The stiffness and surface density are of importance that affects the soundproofing efficiency of single plates [35]. It has been proven that the stiffness is critical below the first resonance frequency, and the material complies with the mass law at higher frequency zones. The STL of material increases with the increase of the stiffness and surface density. In order to identify the factors that dependents to sound insulation property of TPR composites, the potential parameters are listed in Table 2. It has been reported that the presence of CaCO_3_ or HGM in the matrix could restrict the movement of molecules that would lead to the higher elastic modulus and stiffness [36,37,38]. Compared to neat TPR sample, the stiffness and surface density of TPR composite samples are improved by at least 70% and 4%, respectively. With the increment of CaCO_3_ content, the stiffness and surface density increased, leading to the improved sound insulation property and the shifting of the resonance frequency moving to high frequency region. However, the surface density of HGM-filled composites was lower to that of CaCO_3_-filled composites at the same filler content. It is difficult to explain the improved STL value of HGM-filled composites by using the parameters that dominate the STL of single plate.

**Figure 4:**
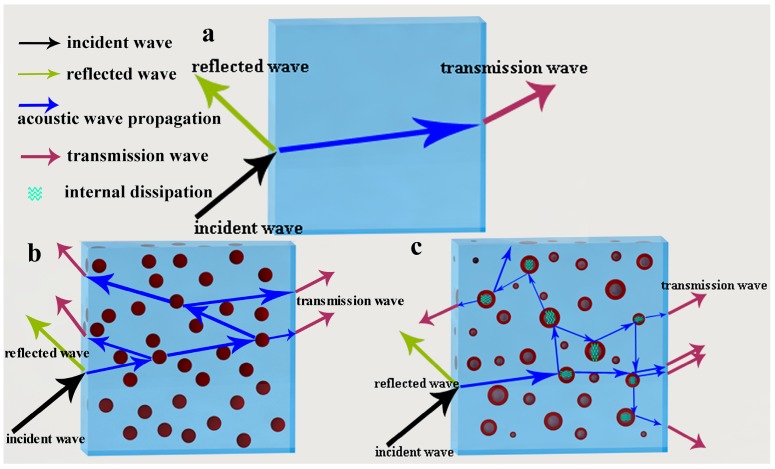
The possible mechanism of the dissipation and damping of sound wave pathway in (**a**) neat TPR, (**b**) TPR/CaCO_3_ and (**c**) TPR/HGM composites.

In consider with the hollow structure of HGM particles and the density of HGM is little greater than that of polymer, sound insulation property of HGM-filled composite is relative to the material density, the content and size of the filler particles, sound speed as well as the sound frequency [39,40]. Acoustic impedance, which depends on the density and sound speed, can be modified by adding filler [41]. Table 2 summarizes the relative sound speed and acoustic impedance of various composites. The acoustic impedance mismatch of neat TPR and its composites were induced by the heterogeneous dispersion of “hard” inorganic particles in “soft” TPR matrix. As shown in Figure 4, when an incident sound wave propagated through a material, the sound energy of incident wave will be transferred into three parts, energy of reflected wave, energy dissipated by material and energy of transmitted wave. Firstly, the damping property of the TPR matrix could absorb the mechanical vibration energy, thus the matrix could dissipate some of the sound energy. Secondly, the sound propagation routine through the composite also plays an important role. The interfaces between the hard particles and the TPR matrix, and the interfaces between HGM and inner air could scatter, diffract and refract sound waves energy. Thirdly, the air inside the inner cavity of the HGM was confined in a narrow space and could dampen the sound waves in the composite [42].

**Figure 5:**
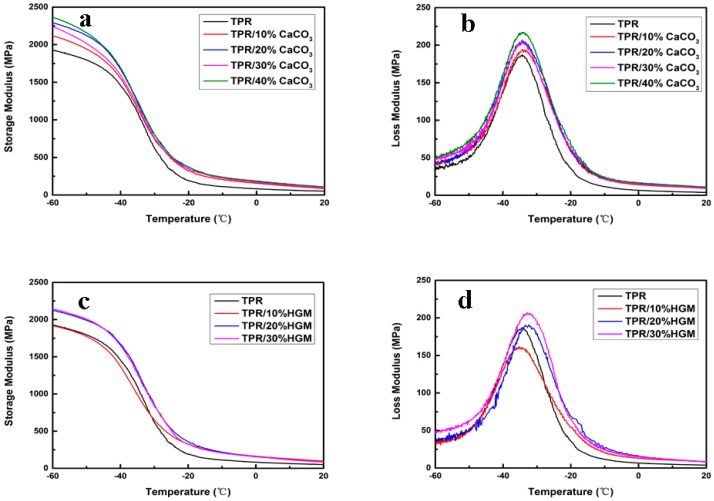
(**a**) Storage modulus (*E*′) of TPR/CaCO_3_ composites; (**b**) loss modulus (*E*″) of TPR/CaCO_3_ composites; (**c**) storage modulus (*E*′) of TPR/HGM composites; (**d**) loss modulus (*E*″) of TPR/HGM composites.

The acoustic energy dissipation of material was associated with loss modulus and tan *δ* of the material [43,44,45,46]. In this study, the loss modulus measured by DMA was used to characterize the vibration damping of TPR composites, which could represent the energy dissipating ability of material. The storage modulus and loss modulus as function of temperature were shown in Figure 5. It is clear that the storage modulus (*E*′) of TPR composites showed great improvement compared with that of virgin TPR sample in the whole tested temperature range (Figure 5a,c). It indicates the enhanced stiffness by the addition of inorganic fillers, which consists with the aforementioned discussion. The loss modulus (*E*″) of TPR composites was also improved (Figure 5b,d). It means TPR composites could dampen more mechanical vibration and dissipate more acoustic energy during the sound propagation in the composites. With the addition of CaCO_3_ and HGM filler, the peak value of loss factor (tan *δ*) value was slightly decreased (Figure S4). This phenomenon could be due to the phase separation in the TPR matrix. In addition, the heterogeneous particles would constrain the movement of polymer segments.

### 3.4. Mechanical Properties

**Figure 6:**
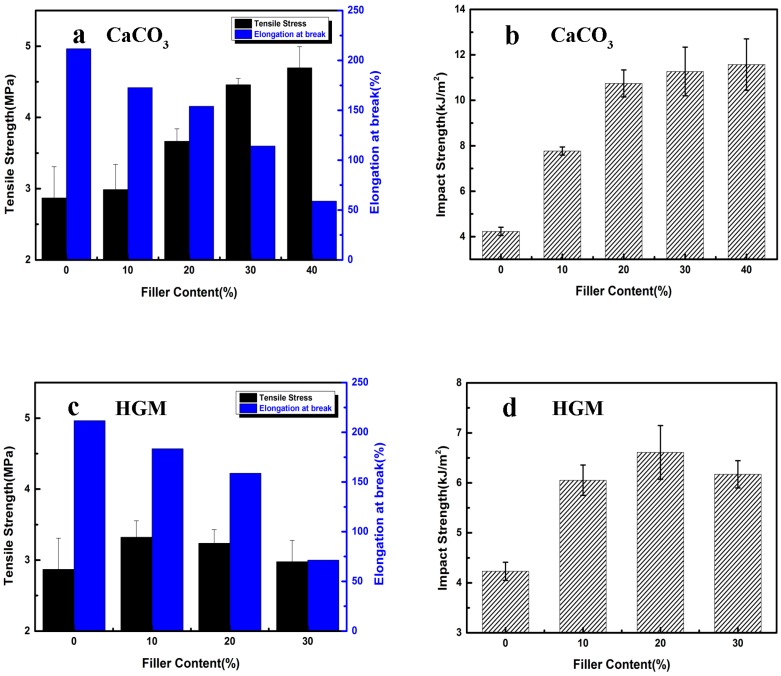
(**a**) Tensile strength of TPR/CaCO_3_ composites; (**b**) impact strength of TPR/CaCO_3_ composites; (**c**) tensile strength of TPR/HGM composites; (**d**) impact strength of TPR/HGM composites.

Beside the sound insulation property, mechanical properties of TPR composites are also important for practical application. The addition of fillers can improve the sound insulation property of the composites, but it may also greatly reduce the mechanical properties of material because of the promoted propagation of cracks in matrix induced by fillers [47]. Figure 6 displays the mechanical property of neat TPR and its composites, and the detailed values are summarized in Table 3. With the increase of the CaCO_3_ content, both the tensile strength and impact strength of composite were obviously increased from the 2.87 MPa of virgin TPR to 4.70 MPa with 40 wt % filler loading, while the elongation at break would be reduced due to restricted polymer chains induced by CaCO_3_ particles. Remarkably, the notched impact strength of neat TPR sample was 4.23 kJ/m^2^ and that of the composite with 20 wt % CaCO_3_ was 10.74 kJ/m^2^, yielding an increase of 153.9%. Compared to CaCO_3_ particles, HGM particles showed an optimal loading for improving the tensile and notched impact strength with 20 wt % HGM loading (Figure 6c,d) [48].

## 4. Conclusions

In this study, the sound insulation performance of TPR materials, especially in low frequency range, was significantly improved after adding either micro-scale CaCO_3_ or HGM particles by simply extrusion method. The all composites showed good processing capability. Owing to well dispersion of fillers in the TPR matrix, the elastic modulus and stiffness of the composite were significantly enhanced. Furthermore, the sound waves pathway through the composite propagated much longer, resulting in more refraction and dissipation of sound energy. In addition, composites showed good damping capability for mechanical vibration compared to that of virginal TPR. Besides, the mechanical properties of composites were obviously improved in the aide of the well-dispersed particles. This study provides an alternative and feasible approach for industry-scale production of TPR based soundproof materials.

## Figures and Tables

**Table 1:** The detailed specification of thermoplastic rubber (TPR) based composites.

Sample	Description
TPR	Melt blended PP/EPDM TPR (PP/EPDM = 30/70)
TPR/10%CaCO_3_	TPR composite with 10 phr CaCO_3_
TPR/20%CaCO_3_	TPR composite with 20 phr CaCO_3_
TPR/30%CaCO_3_	TPR composite with 30 phr CaCO_3_
TPR/40%CaCO_3_	TPR composite with 40 phr CaCO_3_
TPR/10%HGM	TPR composite with 10 phr HGM
TPR/20%HGM	TPR composite with 20 phr HGM
TPR/30%HGM	TPR composite with 30 phr HGM

PP/EPDM: Melt blending of PP with EPDM (PP/EPDM = 30/70); HGM: hollow glass microspheres.

**Table 2:** Mechanical and acoustic parameters of PP, EPDM (Ethylene propylene diene monomer) and different TPR composites.

Sample	Density (10^3^ kg/m^3^)	Surface Density (kg/m^2^)	Elastic Modulus (MPa)	Poisson Ratio	Stiffness (10^−2^ Nm)	Sound Speed (m/s)	Acoustic Impedance (10^3^ Pas/m)
TPR	0.87	4.36	50.73	0.30	54.30	279.47	243.42
TPR/10%CaCO_3_	0.90	4.50	86.12	0.31	92.54	365.71	329.14
TPR/20%CaCO_3_	0.96	4.82	104.14	0.35	113.31	415.88	400.50
TPR/30%CaCO_3_	1.06	5.30	96.90	0.40	107.71	438.99	464.89
TPR/40%CaCO_3_	1.15	5.73	108.40	0.31	116.38	362.24	414.77
TPR/10%HGM	0.89	4.42	97.97	0.30	104.94	387.50	342.28
TPR/20%HGM	0.90	4.50	83.83	0.32	90.37	367.22	330.32
TPR/30%HGM	0.94	4.72	85.64	0.31	91.81	352.00	332.22

**Table 3:** The mechanical properties of TPR composites.

Sample	Tensile Strength (MPa)	Elongation at Break (%)	Impact Strength (KJ/m^2^)
TPR	2.87 ± 0.44	211.70 ± 2.32	4.23 ± 0.18
TPR/10%CaCO_3_	2.99 ± 0.35	172.75 ± 3.36	7.765 ± 0.18
TPR/20%CaCO_3_	3.67 ± 0.18	154.02 ± 2.25	10.74 ± 0.59
TPR/30%CaCO_3_	4.46 ± 0.09	114.23 ± 3.49	11.27 ± 1.07
TPR/40%CaCO_3_	4.70 ± 0.30	59.00 ± 3.55	11.57 ± 1.13
TPR/10%HGM	3.32 ± 0.23	183.51 ± 3.42	6.05 ± 0.30
TPR/20%HGM	3.24 ± 0.19	158.85 ± 3.69	6.61 ± 0.54
TPR/30%HGM	2.98 ± 0.30	71.47 ± 4.01	6.17 ± 0.27

## Supplementary Materials

The following are available online at http://www.mdpi.com/2073-4360/10/3/276/s1, Figure S1: The average STL value of different TPE composites, Table S1: Mechanical and acoustic parameters of different TPE composites, Figure S2: Cut-away diagram of the transmission loss tube, Figure S3: The loss factor curves of TPR materials with different EPDM content, Figure S4: The loss factor curves of (a) TPR/CaCO3 and (b) TPR/HGM composites.
