# Supplementary material for: Morphological Structure, Rheological Behavior, Mechanical Properties and Sound Insulation Performance of Thermoplastic Rubber Composites Reinforced by Different Inorganic Fillers
Source: Polymers (Basel). 2018 Mar 7;10(3):276. doi: 10.3390/polym10030276 (PMC6414929; doi:10.3390/polym10030276)
Supplement: Supplementary file 1 [file polymers-10-00276-s001.docx]

Article

Morphological Structure, Rheological Behavior, Mechanical Properties and Sound Insulation Performance of Thermoplastic Rubber Composites Reinforced by Different Inorganic Fillers

Yanpei Fei ^1^, Wei Fang ^1^, Mingqiang Zhong ^1^, Jiangming Jin ^2,^*, Pin Fan ^1^, Jingtao Yang ^1^, Zhengdong Fei ^1^, Feng Chen ^1,^*, Tairong Kuang ^3,4,^*

^1^ College of Materials Science and Engineering, Zhejiang University of Technology, Hangzhou 310014, China; 201101391305@zjut.edu.cn (Y.F.); 2111625018@zjut.edu.cn (W.F.); zhongmq@zjut.edu.cn(M.Z.); fanping@zjut.edu.cn(P.F.); yangjt@zjut.edu.cn(J.Y.); feizd@zjut.edu.cn(Z.F.)

^2^ College of Mechanical Engineering, Zhejiang University of Technology, Hangzhou 310014, China

^3^ Key Laboratory of Polymer Processing Engineering of Ministry of Education, South China University of Technology, Guangzhou 510630, China

^4^ State Key Laboratory of Molecular Engineering of Polymers, Fudan University, Shanghai 200433, China

***** Correspondence: jjm@zjut.edu.cn (J.J.); chenf@zjut.edu.cn (F.C.); kuangtr@scut.edu.cn (T.K.)

Supplementary Information

1. Characterization of neat TPE and composites


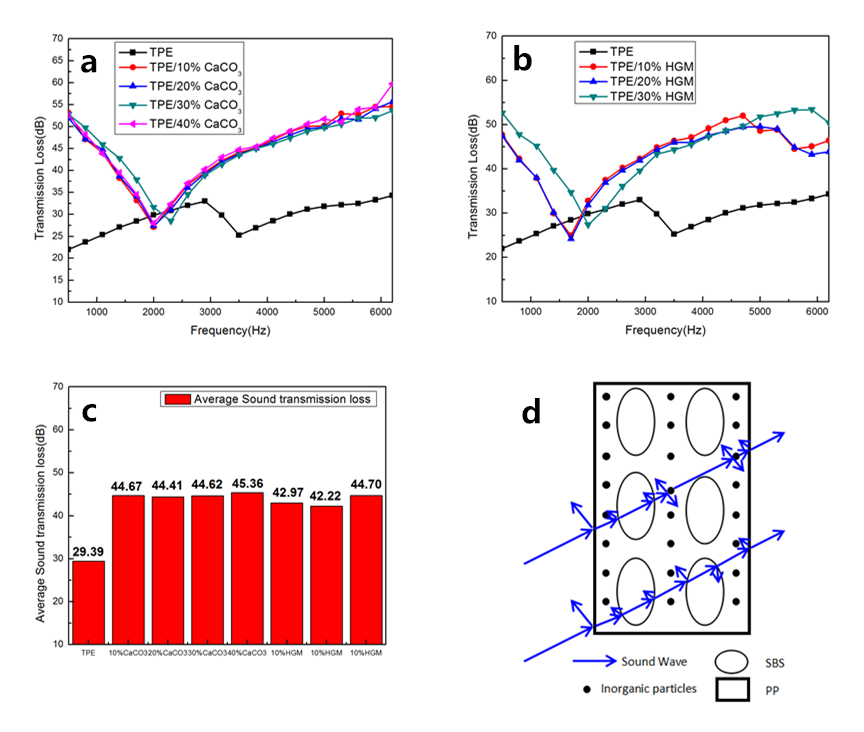


**Figure S1.** The average STL value of different TPE composites.

**Table S1.** Mechanical and acoustic parameters of different TPE composites.

| Sample | Density  (10^3^ kg/m^3^) | Surface Density  (kg/m^2^) | Elastic Modulus  (MPa) | Poisson Ratio | Stiffness  (10^-2^ Nm) | Sound Speed  (m/s) | Acoustic Impedance  (10^3^ Pas/m) |
| --- | --- | --- | --- | --- | --- | --- | --- |
| TPE | 0.934 | 4.670 | 37.108 | 0.385 | 40.986 | 276.604 | 258.348 |
| TPE/10%CaCO_3_ | 1.023 | 5.115 | 47.477 | 0.353 | 51.729 | 274.685 | 281.003 |
| TPE/20%CaCO_3_ | 1.078 | 5.390 | 87.771 | 0.350 | 95.513 | 361.252 | 389.429 |
| TPE/30%CaCO_3_ | 1.098 | 5.490 | 95.786 | 0.333 | 103.613 | 361.717 | 397.165 |
| TPE/40%CaCO_3_ | 1.113 | 5.565 | 63.121 | 0.323 | 68.035 | 286.125 | 318.457 |
| TPE/10%HGM | 0.904 | 4.520 | 70.935 | 0.368 | 77.780 | 370.952 | 335.341 |
| TPE/20%HGM | 0.827 | 4.135 | 66.779 | 0.353 | 72.765 | 362.489 | 299.778 |
| TPE/30%HGM | 0.776 | 3.880 | 64.403 | 0.339 | 69.811 | 356.842 | 276.911 |

2. Sound Transmission Loss Method

Four microphones measure the sound pressure in source tube and receiving tube ^[1]^. As we know the spacing between the microphones and the distance, it is possible to separate the incident and reflected waves by calculated the transfer function of four microphones. The transmission coefficient can be calculated as the ratio between incident sound pressure in the source tube and transmitted sound pressure in the receiving tubes. The transmission coefficient can be calculated as the ratio between incident sound pressure in the source tube and transmitted sound pressure in the receiving tubes ^[2]^.

| $\left\{\begin{matrix} A_{1}\left(\omega\right) \\ B_{1}\left(\omega\right) \end{matrix}\right\}=\left\{\begin{matrix} \alpha\left(\omega\right) & \beta\left(\omega\right) \\ \gamma\left(\omega\right) & \delta\left(\omega\right) \end{matrix}\right\}\left\{\begin{matrix} A_{2}\left(\omega\right) \\ B_{2}\left(\omega\right) \end{matrix}\right\}$ | (1) |
| --- | --- |

Incident Wave:

| $A_{1}\left(\omega\right)=\alpha\left(\omega\right)A_{2}\left(\omega\right)+\beta\left(\omega\right)B_{2}\left(\omega\right)$ | (2) |
| --- | --- |

Transmitted Wave:

| $B_{1}\left(\omega\right)=\gamma\left(\omega\right)A_{2}\left(\omega\right)+\delta\left(\omega\right)B_{2}\left(\omega\right)$ | (3) |
| --- | --- |

So, the sound transmission loss (STL) is:

| $STL\left(\omega\right)=20\log_{10}\left\vert\alpha\left(\omega\right)\right\vert$ | (4) |
| --- | --- |

As the equation listed above can’t be solved by one measure, at least two measurements with two different acoustic terminators is necessary to determinate the transmission coefficients. And the more acoustic properties of absorbent are different, the more accuracy results we can get.

The scheme of the measurement is shown in Fig. S1 The sample is in the middle of the tube, the sound wave is produced by the speaker, and four microphones are used to measure the sound power. As shown in Fig. S1 the “**1**” is the incident sound energy, the “**3**” is the transmitted sound energy, and the “**2**” is the reflected sound energy.

**Figure S2.** Cut-away diagram of the transmission loss tube.

3. Characterization of neat TPR and composites


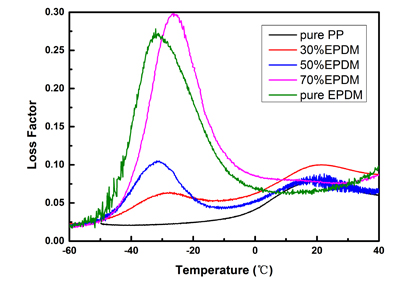


**Figure S3.** The loss factor curves of TPR materials with different EPDM content.

`
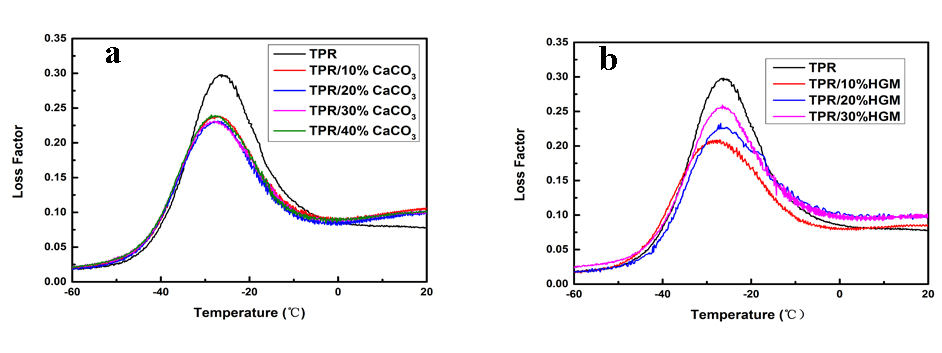


**Figure S4.** The loss factor curves of (a) TPR/CaCO_3_ and (b) TPR/HGM composites.

References

1. User Manual: Impedance/Transmission Loss Measurement Tubes Type 4206, Bruel and Kjaer (2010).
2. Olivieri, J. S. Bolton and T. Yoo. Measurement of transmission loss of materials using a standing wave tube. Proceedings of INTER-NOISE 2006. Honolulu, Hawail, USA (2006).
